# Highly Calibrated Relationship Between Bleomycin Concentrations and Facets of the Active Phase Fibrosis in Classical Mouse Bleomycin Model

**DOI:** 10.3390/ijms252212300

**Published:** 2024-11-15

**Authors:** Anil Hari Kadam, Jan E. Schnitzer

**Affiliations:** Proteogenomics Research Institute for Systems Medicine (PRISM), 505 Coast Blvd. South, La Jolla, CA 92037, USA; kadamanilh@gmail.com

**Keywords:** active phase fibrosis, bleomycin, pulmonary fibrosis, in vivo readouts, biomarkers, facets of experimental fibrosis, fibrotic phenotype, improved accuracy, in vivo therapeutic intervention(s)

## Abstract

The mouse bleomycin model is useful in pre-clinical IPF research to understand pathophysiological mechanisms and pharmacological interventions. In the present study, we systematically investigated the effects of bleomycin at a 60-fold dose range on experimental features of lung fibrosis in the mouse bleomycin model. We analyzed the effect of intratracheal (i.t.) dosing of 0.05–3 U/kg bleomycin on disease phenotypes, including weight loss, morbidity and mortality, pulmonary inflammation, lung collagen content, various BALF biomarkers, and histology in a 14-day mouse model when the animals are in the active phase of fibrosis. In mice, challenge with 1–2 U/kg bleomycin doses induced significant and saturated responses on fibrotic endpoints, confirmed by collagen content, BALF biomarker levels, and marked weight loss compared to the normal control (NC). We observed 100% mortality in 3 U/kg of bleomycin-treated mice. In contrast, 0.05–0.5 U/kg bleomycin doses induced a dose-dependent fibrotic phenotype. The mice challenged with doses of 0.25–0.5 U/kg bleomycin showed optimum body weight loss, a significant increase in pulmonary inflammation, and the fibrotic phenotype compared to NC. Furthermore, we showed 0.25–0.5 U/kg bleomycin increases expression levels of (pro-) fibrotic cytokines, which are the mediators involved in the activation of myofibroblast during fibrogenesis (TGF-β1, IL-13, IL-6, WISP-1, VEGF), angiogenesis (VEGF), matrix remodeling (TIMP-1), and non-invasive lung function biomarker (CRP) compared to NC. A modified Ashcroft scale quantified that the fibrotic changes in the lungs were significantly higher in the lung of mice dosed at 0.25–0.5 U/kg > 0.1 U/kg bleomycin and non-significant in mice lung dosed at 0.05 U/kg bleomycin compared to NC. We demonstrated that the changes due to 0.25–0.5 U/kg i.t. bleomycin on protein biomarkers are enough to drive robust and detectable fibrotic pathology without mortality. The 0.1 U/kg has a moderate phenotype, and 0.05 U/kg had no detectable phenotype. The Goodness of Fit (*r*^2^) and Pearson correlation coefficient (*r*) analyses revealed a positive linear association between change evaluated in all experimental features of fibrosis and bleomycin concentrations (0.05–0.5 U/kg). Here, we provide an examination of a highly calibrated relationship between 60-fold bleomycin concentrations and a set of in vivo readouts that covers various facets of experimental fibrosis. Our study shows that there is a dose-dependent effect of bleomycin on the features of experimental fibrosis at <1 U/kg, whereas saturated responses are achieved at >1 U/kg. Our careful experimental observations, accuracy, and comprehensive data set provided meaningful insights into the effect of bleomycin dose(s) on the fibrotic phenotype, which is valuable in preclinical drug development and lung fibrosis research. In addition, we have presented a set of reproducible frameworks of endpoints that can be used for reliable assessment of the fibrotic phenotype, and in vivo therapeutic intervention(s) with improved accuracy.

## 1. Introduction

Idiopathic pulmonary fibrosis (IPF) is a devastating, progressive disease marked by excessive scarring, which leads to increased tissue stiffness, loss of lung function, and, ultimately, death. IPF is characterized by progressive fibroblast and myofibroblast proliferation and extensive deposition of extracellular matrix (ECM) [1], resulting in abnormal remodeling of the lung parenchyma. The fibrotic triggers in IPF are unknown, but it is speculated that persistent lung injury leads to alveolar epithelial cell injury and death, and subsequent epithelial progenitor exhaustion and/or dysfunction lead to disrepair mechanism(s) that ablate the alveolus [1].

Animal models are essential for successful clinical translation of potential therapeutics. More appropriate preclinical testing in fit-for-purpose models would result in increased clinical success rates for drugs in development [2]. A variety of animal models of pulmonary fibrosis have been developed to study IPF human disease by administration of (a) chemical agents, e.g., bleomycin; (b) inorganic particles, e.g., asbestos, silica, and fluorescein isothiocyanate (FITC) directly into the lung; and (c) overexpression of cytokines, including TGF-β (transforming growth factor beta 1), IL-13 (interleukin-13), TNF-α (tumor necrosis factor-alpha), IL-1β (interleukin-1beta), and others, and using gene-transfer or transgenic approaches [3]. Among all, the bleomycin model of pulmonary fibrosis is the best-characterized rodent model. Bleomycin is an antineoplastic agent used in the treatment of several malignancies, and lung toxicity is one of the major adverse drug effects in human cancer therapy [4]. It is known to cause DNA damage and inhibit DNA synthesis, generating free radicals and leading to inflammation, necrosis, and apoptosis. Its commonly observed adverse effect of lung toxicity is used to induce inflammation and lung fibrosis in rodents [5,6,7,8]. It has been reported that intratracheal administration of bleomycin leads to lung patchy parenchymal inflammation, epithelial cell injury with reactive hyperplasia, epithelial–mesenchymal transition, activation and differentiation of fibroblasts to myofibroblasts, and basement membrane and alveolar epithelium injuries [9]. Bleomycin induces epithelial cell death, followed by an excessive inflammatory response with the release of soluble inflammatory and profibrotic mediators in the early phase. This generates fibroblast activation, ECM deposition, and the development of fibrosis at the molecular and histologic levels, which occur in the late phase [8]. Time points in response to bleomycin revealed an inflammation phase (days 1–2), an active fibrosis phase (days 7–14), and a late fibrosis phase (days 21–35) in rodents [10]. The inflammation phase was largely characterized by the presence of neutrophils in the airways and the increased expression of immune processes in the lung tissue [7]. The acute response rapidly diminished with time and was followed by a sustained increase in lymphocytes and macrophages (days 7 to 21) [11]. Lymphocytes are capable of generating fibroblast chemotactic factors and factors that influence both fibroblast proliferation and collagen synthesis [11]. The active fibrosis phase of the response to bleomycin is characterized by increased levels of (protein and lipid) profibrotic mediators associated with increased matrix deposition in the lung and alterations in pulmonary mechanics [4,10,11]. Reports indicate bleomycin drives progressive fibrosis in rodents by inducing increased expression of pro-inflammatory/profibrotic cytokines such as in IL-1β, TNF-α, interleukin-6 (IL-6), interferon-γ (INF-γ), TGFβ-1, cytokine-induced neutrophil chemoattractant-1 (CINC-1), vascular endothelial growth factor (VEGF), WNT1-inducible-signaling pathway protein 1 (WISP-1), and a tissue inhibitor of metalloproteinases-1(TIMP-1), IL-13, CRP (C-reactive protein), α-SMA (alpha-smooth muscle actin), CTGF (connective tissue growth factor), vimentin, fibronectin, procollagen-1A1, and procollagen-3A1 [4,7,8,10,11,12,13,14,15]. The fibrotic changes reported maximally increased at 14 days after bleomycin instillation, and there was no further increase from 14 days to 21 days [10,11,12,13]. Furthermore, an increased expression of principle pathways/components such as the TGFβ1/Smad pathway, platelet-derived growth factor (PDGF)/AKT pathway, galectin-3, transglutaminase-2, lysophosphatidic acid-1 (LPA-1), and lysyl oxidase-like- 2 (LOXL-2) have been reported in the bleomycin model and human IPF as critical contributors in driving fibrogenesis [8,15,16]. These reports indicate that the bleomycin model has been studied extensively, and precise reports have been made on the time-dependent changes and fibrotic disease mechanisms post-administration of bleomycin. Also, a growing number of preclinical studies have identified promising therapeutic options in rodents [9,10,11,12,17].

Single bleomycin instillation effectively replicates several of the specific pathogenic molecular changes associated with IPF and is used as a model for patients with active disease [10]. This model also has limitations, such as the involvement of a profound initial inflammatory phase, heterogenous distribution of fibrosis, and spontaneous reversal of fibrosis [18]. Despite these limitations, Pirfenidone, the first clinically approved IPF therapy, followed by recently approved Nintedanib, has been extensively evaluated in bleomycin–rodent models, e.g., hamsters, mice, and rats [19,20,21,22,23,24,25,26], and many other potential therapies also evaluated in this model are in advanced stages of clinical development [27]. These studies highlight the translational application of the bleomycin rodent model to validate the common mechanistic pathways driving disease pathogenesis in IPF patients and potential responses to therapy [4].

Most published studies use mice as the species of choice, the intratracheal (i.t.) route of administration, and single doses of bleomycin ranging from 1.25 to 4 U/kg. The dose range of 2 to 2.5 U/kg provides a more effective model. The development of the disease is rapid, with inflammation peaking at around 7 days and the development of fibrosis (day 10–21 with a peak around day 14) at the molecular and histology levels following bleomycin administration [17]. In preclinical fibrosis research, optimized experimental conditions and a controlled fibrotic disease phenotype enhance the accuracy and reliability of assessing the phenotype, underlying causes of pulmonary fibrosis efficacy, and responses to experimental therapeutics that advance IPF research and drug development efforts [8]. Furthermore, disease model refinement can be achieved by studying the effect of the range of bleomycin concentrations on the fibrotic phenotype. In previous studies, dose–response effects of 1, 2, and 4 mg/kg bleomycin in a mouse model have been reported by Kim et al., 2010, where the endpoint analysis was limited to body weight loss, inflammatory cell analysis, levels of lactate dehydrogenase (LDH), and histology [28]. Another report by Jean-Claude Gilhodes et al. reported dose effects of 0.25, 0.5, 0.75, and 1 mg/kg i.t. bleomycin, providing details of improved automated histological image analysis, fibrotic Ashcroft scoring, body weight loss, and lung weight readouts [29]. Given the popularity and utility of the mouse bleomycin model, no published report has systematically examined an expanded array of features of experimental fibrosis using a wider range of bleomycin doses.

We report here the result of a study conducted in mice using i.t. administration of bleomycin doses ranging from 0.05 to 3 U/kg. We examined the relationship between 60-fold bleomycin concentrations and a set of in vivo readouts that covers various facets of experimental fibrosis. Our data provide an understanding of the effect of bleomycin dose(s) on preclinical (in vivo) endpoints, such as clinical signs, pulmonary inflammation, lung parameters, biomarkers involved in the activation of myofibroblast, vascular permeability, angiogenesis, extracellular tissue remodeling, non-invasive lung function, and histology. The data and investigation suggest that bleomycin <1 U/kg provides a controlled experimental fibrotic phenotype compared to >1 U/kg bleomycin doses. Using <1 U/kg bleomycin doses, we identify 0.25–0.5 U/kg as the ideal bleomycin concentrations that induced a robust fibrotic phenotype without mortality, and also provide the possible mechanism(s) attributed to the fibrotic phenotype due to these concentrations. This report’s data findings and detailed analysis insights in a classical preclinical model will be useful in model refinement and experimental design, and invaluable in the reliable assessment of facets of experimental lung fibrosis and the efficacy of future IPF lead molecules.

## 2. Results

We assessed the dose effect of 0.05–3 U/kg bleomycin on clinical signs such as body weight loss and mortality from day 0 to 14. Animal deaths were observed in the bleomycin treatment at 3 U/kg, with no survivors until day 14. Therefore, we evaluated inflammation, lung parameters, and BALF fibrotic biomarkers following administration of 0.05–2 U/kg bleomycin in mice, as described in Section 4.

### 2.1. Bleomycin Greater than 1 U/kg Induces Saturated Fibrotic Phenotype

We noticed bleomycin 100% mortality in groups of mice dosed with 3 U/kg (100%) (Supplementary Figure S1). Weight loss was approximately 20% and >30%, respectively, in bleomycin 1–2 U/kg- and 3 U/kg-treated mice towards the end of the study (Supplementary Figure S1). After 14 days, bleomycin 1–2 U/kg-treated mice exhibited significantly (*p* < 0.05, *p* < 0.001) increased numbers of total leukocyte cells, neutrophils, lymphocytes, and macrophages in BAL fluid compared to NC (Supplementary Figure S2A–D). Similarly, lung parameters such as lung collagen content, lung weight, lung index readouts, and various BALF biomarkers, including protein content, TGF-β1, IL-6, VEGF, TIMP-1, and CRP, were significantly (*p* < 0.05, *p* < 0.001) increased in bleomycin 1–2 U/kg-treated mice 14 days post-treatment (Supplementary Figures S3A–C and S4A–F). In bleomycin 1–2 U/kg treatment, there was a similar degree of collagen content and all BALF biomarker readouts changes, whereas mortality was observed with progressive weight loss in 3 U/kg (Figure 4 and Supplementary Figures S1–S5). This directed us to test lower doses of bleomycin in the next study. We tested the effect of 0.05–0.5 U/kg bleomycin on clinical signs, inflammation, collagen content, and BALF biomarkers.

### 2.2. Bleomycin Less than 1 U/kg Induces Dose-Dependent Weight Loss Without Mortality

**Figure 1:**
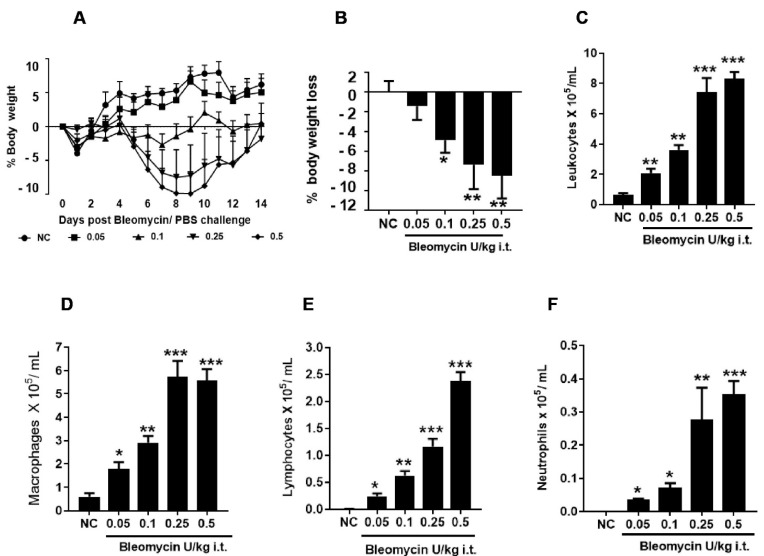
Effect of i.t. bleomycin concentrations on clinical signs and pulmonary inflammation. (**A**) % body weight, (**B**) % body weight loss, (**C**) leukocytes, (**D**) macrophages, (**E**) lymphocytes, (**F**) neutrophils. Data are expressed as mean ±SEM of n=5 mice/group. * *p* < 0.05; ** *p* < 0.01; and *** *p* < 0.001 Vs NC.

A single i.t. challenge in mice with 0.1, 0.25, and 0.5 U/kg bleomycin induced significant body weight loss (*p* < 0.05), but this was not evident in mice dosed at 0.05 U/kg bleomycin, as compared to NC (Figure 1A,B). In mice dosed at 0.25 and 0.5 U/kg, body weight loss was progressive until day 7, at which point it stabilized between days 7 to 10, followed by daily weight increases until day 14. In contrast, mice challenged with 0.05 and 0.1 U/kg bleomycin did not exhibit progressive body weight loss but were ranked 0.1 U/kg > 0.05 U/kg (Figure 1A,B). The % body weight loss was noted of −1.4%, −4.84%, −7.32%, and −8.46% in a dose-dependent manner in mice dosed at 0.05, 0.1, 0.25, and 0.5 U/kg bleomycin, respectively (Figure 1B). No mortality was observed in bleomycin-dosed mice and NC. In this study, we did not observe severe weight loss, reduced physical activity, shallow breathing, or a moribund state after the bleomycin challenge, reflecting the satisfactory physiological state of animals.

### 2.3. Bleomycin Less than 1 U/kg Induces Dose-Dependent Pulmonary Inflammation

The BALF analysis for inflammation reveals a dose-dependent 3–14-fold increase in total leukocyte count, which was markedly significant (*p* < 0.01) in mice dosed at 0.05–0.5 U/kg bleomycin (Figure 1C and Supplementary Figure S5). Similarly, macrophages significantly increased (*p* < 0.05, *p* < 0.001) in mice treated at 0.05–0.5 U/kg bleomycin (Figure 1D and Supplementary Figure S5). The lymphocytes increased in a dose-dependent manner, which was most significant (*p* < 0.01) between doses of 0.1 and 0.5 U/kg bleomycin (Figure 1E and Figure S5). A similar pattern was observed for the neutrophil increase and was found to be significant (*p* < 0.01) at 0.25 and 0.5 U/kg (Figure 1F and Figure S5). The increases in leukocytes, macrophages, lymphocytes, and neutrophils were more modest in animals dosed at 0.05 and 0.1 U/kg bleomycin, whereas their increase was profound in 0.25 and 0.5 U/kg bleomycin-treated animals over NC (Figure 1C–F and Figure S5). The increase in total BALF cells was accounted for by the presence of a majority of macrophages > lymphocytes > neutrophils in mice dosed with bleomycin 0.1–0.5 U/kg; this was in marked contrast with the total count of NC mice, which was comprised solely of macrophages (Figure 1C–F).

### 2.4. Bleomycin Less than 1 U/kg Induces a Dose-Dependent Increase in Vascular Leakiness and Lung Fibrosis

**Figure 2:**
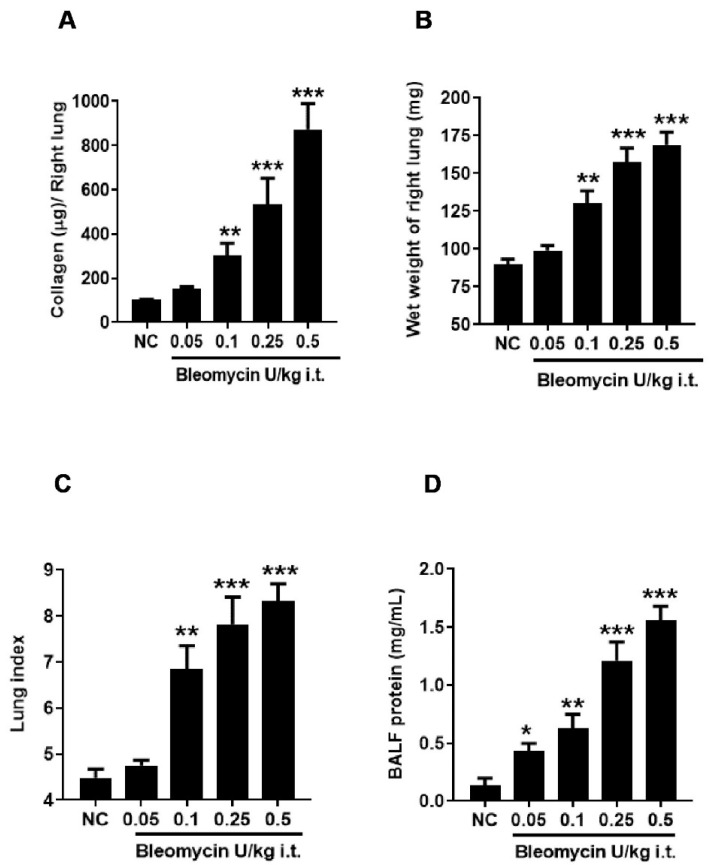
Effect of i.t. bleomycin concentrations on lung parameters and vascular permeability. (**A**) right lung collagen content, (**B**) wet weight of right lung, (**C**) lung index, (**D**) protein content. Data are expressed as mean ± SEM of n=5 mice/group. * *p* < 0.05; **p < 0.01; and *** *p* <0.001 Vs NC.

The lung collagen content significantly (*p* < 0.01, *p* < 0.001) increased in mice dosed at 0.1–0.5 U/kg in a dose-dependent manner compared to NC (Figure 2A and Figure 4A). A similar trend of increase in lung weight and lung index parameters was observed. We noticed a significantly (*p* < 0.01, *p* < 0.001) increased wet weight of the right lung and lung index in mice dosed at 0.1–0.5 U/kg compared to NC (Figure 2B,C and Figure 4B,C). Increased BALF protein content was bleomycin-dose-dependent, and was significantly increased (*p* < 0.05, *p* < 0.001) in mice dosed with 0.05–0.5 U/kg bleomycin compared to NC (Figure 2D and Figure 4D). No significant increase in collagen content, lung weight, or lung index was observed in mice dosed at 0.05 U/kg bleomycin compared to NC (Figure 2A–C and Figure 4A–C).

### 2.5. Bleomycin Less than 1 U/kg Induces a Dose-Dependent Increase of BALF Fibrosis Biomarkers

**Figure 3:**
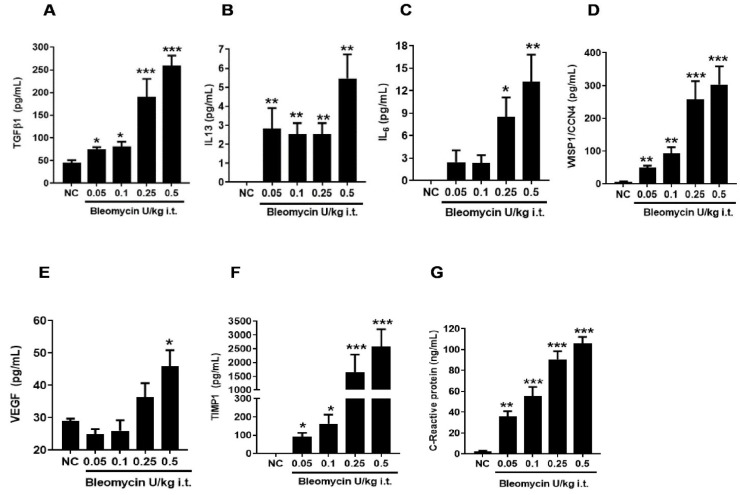
Effect of i.t. bleomycin concentrations on lung fibrosis BALF biomarkers. BALF profibrotic biomarkers (**A**) TGFβ-1, (**B**) IL-13, (**C**) IL-6, (**D**) WISP-1, (**E**) VEGF, (**F**) TIMP-1, and (**G**) CRP. Data are expressed as mean ± SEM of n = 5 mice/group. * *p* < 0.05; ** *p* < 0.01; and *** *p* < 0.001 Vs NC.

**Figure 4:**
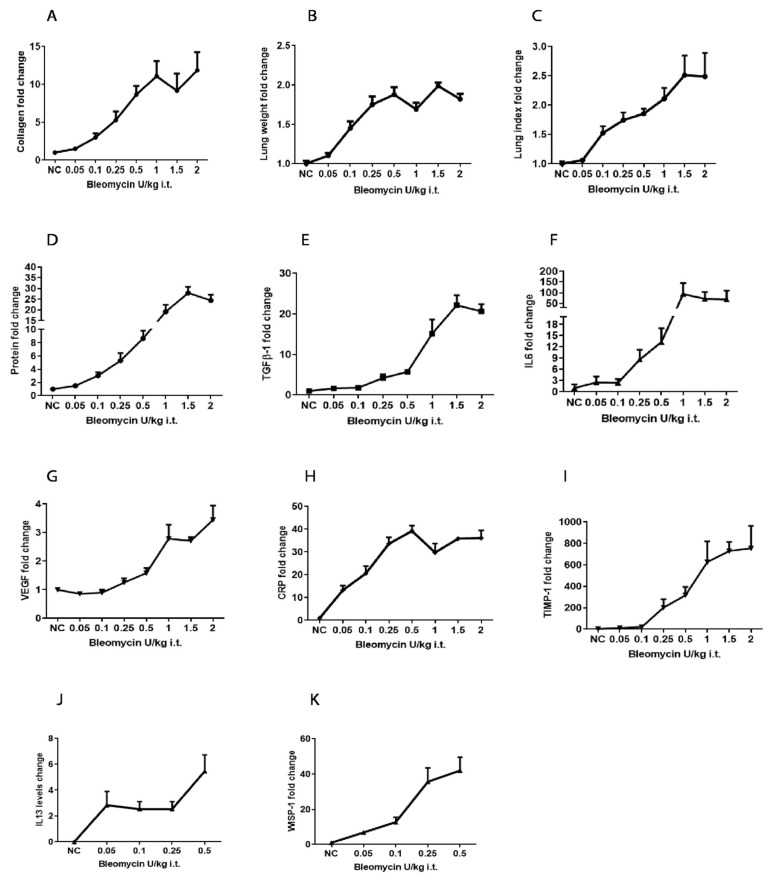
Effect of bleomycin challenge on fold change of Lung parameters and BALF biomarkers. Fold change of (**A**) collagen content, (**B**) lung weight, (**C**) lung index, (**D**) Protein, (**E**) TGFβ-1, (**F**) IL-6, (**G**) VEGF, (**H**) CRP (**I**) TIMP-1 due to 0.05-2 U/kg bleomycin challenge. The levels of (**J**) IL-13*, and fold change of (**K**) WISP-1, due to 0.05–0.5 U/kg bleomycin challenge.

At 14 days following 0.05–0.5 U/kg i.t. bleomycin dosing, we measure levels of profibrotic mediators, e.g., TGFβ-1, IL-13, IL-6, WISP-1, VEGF, TIMP-1, and CRP, present in BALF samples. We noticed that the TGFβ-1, IL-6, WISP-1, VEGF, TIMP-1, and CRP (Figure 3A,C–G and Figure 4E–I,K) levels were elevated in a bleomycin-dose-dependent manner at doses of 0.1–0.5 U/kg, whereas elevated IL-13 levels did not respond according to bleomycin doses (Figure 3B and Figure 4J). Significant (*p* < 0.05) increases in levels of TGFβ-1, IL-13, WISP-1, TIMP-1, and CRP were observed in mice dosed with 0.05–0.5 U/kg bleomycin (Figure 3A,B,D,F,G, respectively) compared to NC. In contrast, IL6 levels significantly increased (*p* < 0.05) in 0.25–0.5 U/kg bleomycin-dosed mice (Figure 3C). The VEGF levels increased significantly (*p* < 0.05) only in mice dosed with 0.5 U/kg bleomycin compared to NC (Figure 3E). The fold change in these biomarkers was noted at 0.5–0.25 U/kg > 0.1 > 0.05 U/kg bleomycin-dosed mice compared to NC (Figure 3A–G and Figure 4E–K).

### 2.6. Bleomycin Less than 1 U/kg Induces Dose-Dependent Fibrotic Changes and Severity in Lung Tissue

**Figure 5:**
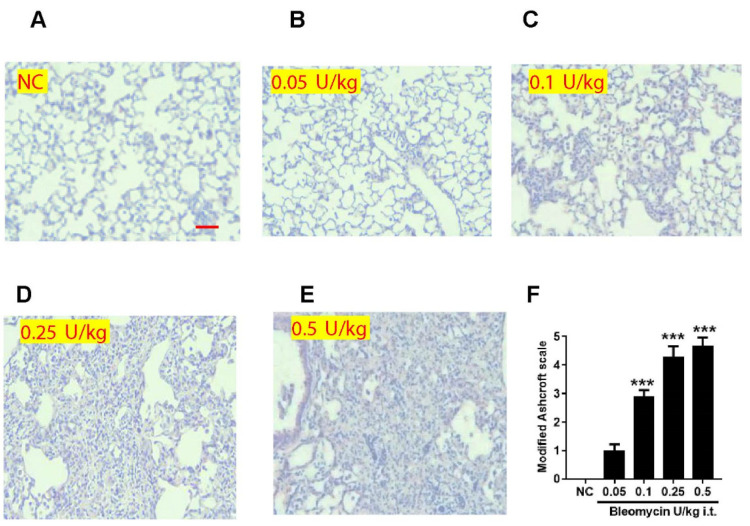
Effect of i.t. bleomycin concentrations on lung pathology and severity of fibrosis. Representative histopathological images (10× magnification) of hematoxylin (H) and eosin (E) staining of mice lung treated with (**A**) PBS; bleomycin (**B**) 0.05, (**C**) 0.1, (**D**) 0.25, (**E**) 0.5 U/kg; severity of fibrosis by Modified Ashcroft scale (**F**). Data are expressed as mean ± SEM of n = 5 mice/group. *** *p* < 0.001 Vs NC. Scale bar:50 µm.

In histological examination of lungs harvested on day 14, the NC and 0.05 U/kg bleomycin-treated animals revealed predominately normal lung architecture (Figure 5A,B). However, in animals dosed with 0.1 U/kg bleomycin, histology revealed clearly profound fibrotic changes with unconnected knot-like formations, contiguous fibrotic walls, and fibrotic masses (Figure 5C). Mice dosed at 0.25 and 0.5 U/kg bleomycin revealed significant infiltration of inflammatory cells, thickening of alveolar septa, damage to the lung tissue, and the deposition of extracellular matrix in lung parenchyma (Figure 5D,E). A modified Ashcroft scale quantified the fibrotic changes in the lungs, which were significantly increased (*p* < 0.001) in the lungs of mice dosed at 0.25–0.5 U/kg (scores between 4 and 5) > 0.1 U/kg bleomycin (score 2.9), but was non-significant in mice lungs dosed at 0.05 U/kg bleomycin with a score of 0.9 by comparison with NC (Figure 5F).

### 2.7. The Severity of Experimental Fibrosis Features Directly Correlates with Bleomycin Concentrations

**Figure 6:**
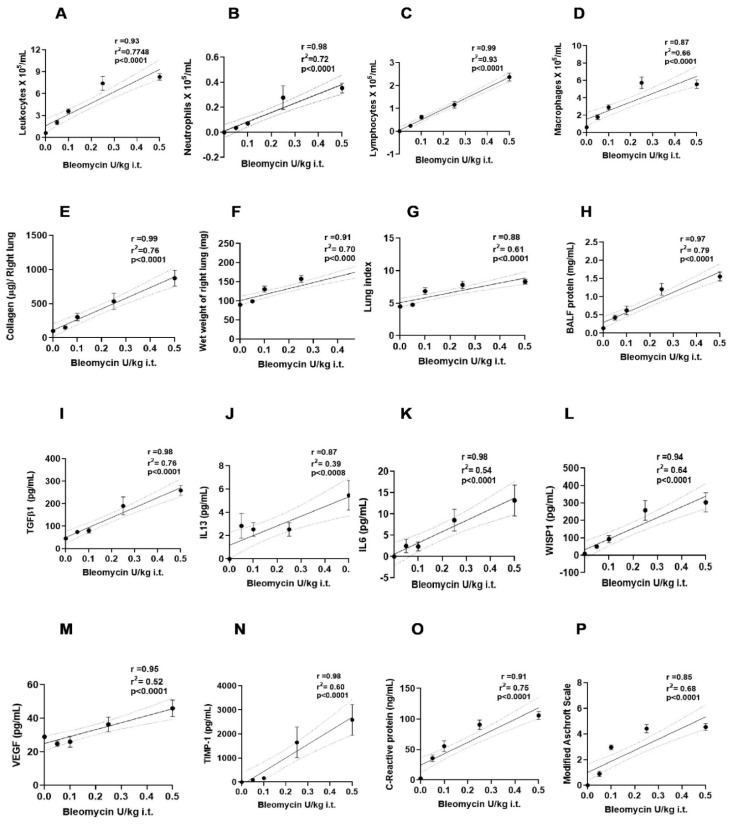
Simple linear regression analysis between concentrations of bleomycin and features of experimental fibrosis. (**A**) leukocytes, (**B**) neutrophils, (**C**) lymphocytes, (**D**) macrophages, (**E**) collagen, (**F**) lung weight, (**G**) lung Index, (**H**) protein content, (**I**) TGFβ-1, (**J**) IL-13, (**K**) IL-6, (**L**) WISP-1, (**M**) VEGF, (**N**) TIMP-1, (**O**) CRP, (**P**) Modified Ashcroft Scale. Goodness of Fit: *r*^2^, Pearson correlation coefficient (*r*), n = 5/group.

The link between concentrations of bleomycin and change in features of experimental fibrosis was analyzed by simple linear regression analysis (Goodness of Fit or coefficient of determination: *r*^2^) and the Pearson correlation coefficient (*r*). Linear regression plots reveal significant (*p* < 0.001) strong positive associations with concentrations of bleomycin in an increase of leukocytes (*r*^2^ = 0.78, *r* = 0.93) neutrophils (*r*^2^ = 0.72, *r* = 0.98), lymphocytes (*r*^2^ = 0.93, *r* = 0.99), and macrophages (*r*^2^ = 0.65, *r* = 0.87) (Figure 6A–D). Similarly, increases in collagen content (*r*^2^ = 0.76, *r* = 0.99), protein content (*r*^2^ = 0.786, *r* = 0.97), lung weight (*r*^2^ = 0.695, *r* = 0.91), and lung index (*r*^2^ = 0.610, *r* = 0.88) were found to be significant (*p* < 0.001) in relation to bleomycin concentrations (Figure 6E–H). The change in the biomarkers TGFβ-1, CRP (*r*^2^ = 0.74–0.75, *r* = 0.91–0.98); WISP-1, VEGF (*r*^2^ = 0.644, *r* = 0.94–0.95); TIMP-1, IL6 (*r*^2^ = 0.596 and 0.538, *r* = 0.98); and IL13 (*r*^2^ = 0.394, *r* = 0.87) was significant (*p* < 0.001) in mice subjected to 0.05–0.5 U/kg bleomycin concentrations (Figure 6I–O). The bleomycin concentrations and lung fibrotic pathology (Ashcroft scores) were noted to be significant (*p* < 0.001) with *r*^2^ = 0.6823, *r* = 0.85 (Figure 6P). The Goodness of Fit (*r*^2^) analysis and correlation analysis (value of *r* found between 0.85 and 0.99) revealed a positive linear association between changes in all experimental features of fibrosis, which were noted to be bleomycin-concentration-dependent (Figure 6A–P).

## 3. Discussion

In the present study, we systematically investigated the effects of various bleomycin doses on experimental features of lung fibrosis in a classical bleomycin model when animals were in the active phase of fibrosis. The highly calibrated result indicated that the clinical signs, inflammatory cellular profiles, lung collagen, lung weight, fibrotic lung environment, and histology changes were related in a bleomycin-dose-dependent manner up to a certain bleomycin concentration without mortality. After reaching higher doses, the changes reached a plateau, and at even higher doses, they led to induced morbidity and mortality.

In our studies, we tested the effect of a total of eight bleomycin concentrations (0.05–3 U/kg) on predetermined endpoints in the mouse bleomycin model [18]. The methods used to administer bleomycin in previous reports are intratracheal, oropharyngeal, intranasal, intraperitoneal, subcutaneous, intravenous, and through osmotic pumps [17,18]. In our study, we choose a commonly used method, i.e., intratracheal, to induce lung fibrosis in mice. The bleomycin model was reported in both male and female C57BL/6J mice. None of the studies failed to induce a fibrotic response regardless of the bleomycin sources and sex used by various investigators (Supplementary Table S1). Furthermore, we also obtained information from published reports on approximately 10 clinical compounds with novel mechanisms that were evaluated in bleomycin-induced lung fibrosis in female C57BL/6 mice. Those compounds are currently successfully advancing through the stages of IPF clinical trials and could be potential novel IPF therapies in the near future if the clinical trials are successful. The compound evaluated in female mice also made progress to clinical stages (Supplementary Table S2). We used female C57BL/6J mice in our study. The investigators successfully used pharmaceutical-grade and chemical-grade bleomycin to induce fibrosis (Supplementary Table S1). We used pharmaceutical bleomycin, a more quality-controlled product used in human treatment. A single dose of bleomycin is sufficient to produce marked histological and biochemical changes in most rodents [28,30], with the peak of fibrosis around day 14 [17]. A similar degree of severity of fibrosis was reported on days 14 and 21 in the mouse model [10], with the 14-day timepoint commonly employed for disease phenotype and therapeutic evaluation [12,24,29]. On day 21 onwards, fibrosis resolution and the standard outcome on parameters are highly variable [4]. Therefore, we choose 14 days (active phase of fibrosis) for endpoint analysis [8].

Investigators reported that bleomycin (1.5–4 U/kg) induces weight loss and mortality in mice [10,31,32]. As expected, our study observed significant body weight loss in mice subjected to 0.1–1 U/kg bleomycin insult without mortality. The weight loss in 1.5, 2, and 3 U/kg bleomycin-dosed mice was marked, severe, and progressive until day 7. In particular, 3 U/kg treatment resulted in mortality. We started observing mortality around day 7 until the end of the experiment. Bleomycin 3 U/kg induced significant 100% mortality in mice, which is consistent with the report of Zhao et al., where they showed 90 % mortality using 2.5 U/kg i.t. bleomycin in a mouse model [33]. In contrast, zero mortality was reported using 1–4 U/kg i.t. bleomycin [29] and 1.5 U/kg bleomycin by oropharyngeal aspiration [34,35]. This could be due to bleomycin’s different sources and potencies, or the route implemented to induce fibrosis in mice. In our data, 3 U/kg doses of bleomycin induced significant mortality, but not 0.05–2 U/kg doses, indicating the mouse bleomycin model is very sensitive to the dose of i.t. bleomycin. The dose should be selected carefully for running experiments, as the success of studies largely depends upon collecting informative data from all animals. Further, mortality represents severe induction of disease, which could mask the pharmacological effect of therapy if the model is used for pharmacological efficacy evaluations.

In the study with 1–3 U/kg bleomycin, further endpoint analysis was performed at 1–2 U/kg bleomycin. We showed a significant increase in pulmonary inflammation and collagen content accompanied by lung weight and index [8]. The BALF fibrosis biomarkers, such as protein content, TGFβ1, IL-6, VEGF, CRP, and TIMP-1, were significantly increased in bleomycin 1–2 U/kg i.t.-treated mice. While the results we obtained using the 1–2 U/kg bleomycin challenge were generally consistent with previous reports [12,36,37,38,39,40,41,42], we noticed saturated effects on endpoints. Therefore, in the next study, we decided to test lower doses at 0.05, 0.1, 0.25, and 0.5 U/kg of bleomycin to achieve a bleomycin-dose-dependent effect of bleomycin on the fibrotic phenotype.

As expected, we observed significant body weight loss in mice dosed at 0.1- 0.5 U/kg bleomycin insult without mortality. Zero death was observed, possibly due to low doses of bleomycin. We noticed an optimum 6–8% body weight loss, recovery from 7–10 days onwards [29,43] at 0.25–0.5 U/kg bleomycin, and weight loss found at 0.1 > 0.05 U/kg. The inflammatory readout revealed a dose-dependent, profound, and significant increase in the order of total leukocytes > macrophages > lymphocytes > and neutrophils at 0.25–0.5 > 0.05–0.1 U/kg in bleomycin-dosed mice [12]. We were unaware of whether tested bleomycin doses elicit a fibrotic response or not. Interestingly, 0.5 > 0.25 > 0.1 U/kg bleomycin induces a significant increase in lung collagen content, accompanied by an increase in lung weight and index, as we observed with 1–2 U/kg bleomycin. This confirms that 0.1–0.5 U/kg bleomycin elicits a fibrotic phenotype. BALF protein content due to bleomycin injury is characteristic of increased vascular permeability and lung damage [12,16]. We showed a dose-dependent increase in BALF protein content from 0.05 to 0.5 U/kg bleomycin, which indicates increased vascular permeability and lung damage.

In addition to phenotypic endpoints resulting from bleomycin insult, we were interested in examining different pathophysiological mechanisms involved in the fibrotic response due to 0.05–0.5 U/kg bleomycin. We analyzed several protein levels of clinically relevant molecular targets and mediators of fibrosis in BALF. Results showed 0.25 and 0.5 U/kg bleomycin increased expression levels of (pro-) fibrotic cytokines, which are the mediators involved in the activation of the myofibroblast during fibrogenesis (TGF-β1, IL-13, IL-6, WISP-1, and VEGF), angiogenesis (VEGF), matrix remodeling (TIMP-1), and biomarker of lung function (CRP) in a mouse model of IPF, which are consistent with previous reports using 1.5–3 U/kg bleomycin [7,8,12,44,45,46,47].

We show increased TGF-β1 levels with bleomycin treatment. Lung fibrosis is associated with increased activity of TGF-β_1_ signaling pathways [48]. TGF-β1, a profibrotic cytokine, plays a pivotal and extensive role in the development of tissue fibrosis in IPF [49,50]. It modulates the transcription of downstream target genes, including procollagen I and III, and SMAD-2/3 signaling pathways [7,51]. Mechanistically, it promotes ECM (extracellular matrix) accumulation, especially collagen and fibronectin, and drives phenotypic changes in fibroblasts [52,53]. In our data, the increase in TGF-β1 suggests that the TGFβ/Smad pathway may be actively driving the fibrotic phenotype at 0.25–0.5 > 0.1–0.05 U/kg bleomycin. In previous studies, TGF-β1 was reported to be profibrotic by up-regulating type I collagen and TIMP-1 expression in fibroblasts [54]. We noticed a parallel increase in TGF-β1 and TIMP-1; this suggests the profibrotic effect of TGF-β1 on TIMP-1 release in vivo is bleomycin-dose-dependent.

In this study, the BALF IL-13 level increase was noted to be similar at 0.05–0.25 doses, which is <0.5 U/kg of bleomycin. The increase in IL-13, the Th2 cytokine, was reported in the blood and BALF of patients with IPF and correlated with disease severity [45]. IL-13 promoted pulmonary fibrosis in radiation-induced lung fibrosis models, whereas IL-13 inhibition decreased fibrotic changes in the IPF model in vivo [55]. Mechanistically, IL-13 activates myofibroblasts through a c-Jun N-terminal kinase-dependent pathway [56]. Downstream IL-13 effects were mediated through a complex receptor system that includes IL-4Ra, IL-13Rα1, and/or IL-13Rα2 [57]. In a recent study, in the bleomycin model, sphingosine-1-phosphate receptor-2 (S1PR2) facilitated pulmonary fibrosis by potentiating the IL-13 pathway in macrophages [58]. In our data, an increase in BALF IL-13 at all doses of bleomycin suggests that the Th2 response and associated functional pathway component may drive a fibrotic phenotype on day 14, in particular at doses of 0.25 and 0.5 U/kg, along with other fibrotic mediators.

We observed a dose-dependent BALF IL-6 level increase, which was significantly observed at 0.25 and 0.5 U/kg. IL-6 is a pleiotropic cytokine and functions as a pro-inflammatory factor and a profibrotic factor in lung fibrosis pathogenesis [59]. IL-6 is expressed and secreted by various cells, including alveolar macrophages, lung fibroblasts, and fibrocytes [60]. Recently, besides TGF-β/Smad3 signaling, the signaling loop of IL-6/gp130/Stat3 has been shown to play a crucial role in the pathogenesis of lung fibrosis [61]. Furthermore, blockade of the IL-6 signal during the chronic stages of lung injury benefits lung fibrosis [62]. These reports suggest the significance of IL-6 in fibrogenesis, and the increase in IL-6 in this study is consistent with previous reports [7,8].

The Wnt-pathway induces epithelial cell proliferation and differentiation, epithelial-to-mesenchymal transition, fibroblast migration, and myofibroblast differentiation [53,63]. Similarly, like TGF-β1, we observed an increase in levels of WISP-1 in all doses of bleomycin, and a profound increase was observed at 0.25 and 0.5 U/kg. Besides TGF-β, dysregulated activation of WISP-1 plays a key role in IPF [64]. The expression of WISP-1, a Wnt target gene, was elevated in AECII cells (alveolar epithelial type II cells) of the mouse bleomycin model and in IPF patients [7,8]. The neutralizing WISP-1 antibody treatment led to the attenuation of lung fibrosis and increased the survival rate in the bleomycin–mouse model, along with reduced collagen deposition and expression of genes associated with EMT (epithelial–mesenchymal transition) [63]. Functionally, WISP-1 mediates IL6-dependent cell proliferation through a mechanism orchestrated by a variety of profibrotic mediators, including Wnts, TGF-β1, and TNF-α in primary human lung fibroblasts [65]. In vivo, increased WNT/β-catenin signaling leads to the expression of the pro-inflammatory cytokines IL-1β and IL-6 in alveolar epithelial type cells in the bleomycin model, and it provides a WNT/interleukin axis link in the development of pulmonary fibrosis [66,67]. In our study data, we have shown parallel increases in levels of IL-6 or TGF-β and WISP1 in BALF, indicating an in vivo WNT/IL-6 or WNT/TGF-β axis link in our model at 0.05 to 0.5 U/kg bleomycin.

Aberrant angiogenesis is also a prominent histopathological feature of IPF, and an approved therapeutic for IPF, Nintedanib, acts by targeting VEGF receptor signaling in combination with inhibiting FGF receptor (fibroblast growth factor receptor) and PDGF receptor (prostaglandin-derived growth factor receptor) activities [8,46,68]. Studies suggest that neovascularization enhances fibrogenesis. In bleomycin-induced lung fibrosis, neutralization of an angiogenic chemokine or administration of an angiostatic chemokine reduced the fibrotic response, paralleled by a reduction in the level of angiogenesis [14]. However, neovascularization is a prominent feature in organizing pneumonia, a usually reversible fibrogenic process [14,69]. We investigated the effect of 0.05 to 2 U/kg bleomycin on VEGF in mice. We noticed that the effect of 0.05 to 0.5 U/kg bleomycin is mild, and 1 to 2 U/kg bleomycin profoundly increases VEGF in BALF. The VEGF levels increased in 1–2 U/kg bleomycin > 0.05–0.5 U/kg. This suggests that bleomycin induces VEGF and associated pathways overall. In a dose-dependent treatment at 0.05–0.5 U/kg, we noticed that only 0.5 U/kg bleomycin might have triggered the release of VEGF during the disease progression, which was sustainable until day 14. In contrast, at 0.05–0.25 U/kg, the kinetic increase in VEGF was not maintained until day 14. Given the role of VEGF in fibrosis resolution, we suggest choosing a dose of bleomycin that induces fibrosis in mice, but it should not trigger the profound release of VEGF in the lung. However, this needs to be validated experimentally.

TIMP-1 was detected in IPF lungs [13,14]. TIMP-l is expressed on macrophages and epithelial cells and is strongly associated with fibrosis. TIMP-1 gene expression and protein are selectively increased in BALF and lung homogenate in the fibrosis model [8,44]. TIMP-1 has also been reported for its important regulatory role in the bleomycin model [70,71]. TIMP1 induces mesenchymal cell proliferation [14]. Several studies have demonstrated that increased expression of TIMP-1 plays a critical role in the development of experimental lung fibrosis [48,72]. In this study, a significant increase in TIMP-1 levels was observed in all doses of bleomycin, which is consistent with a previous report due to bleomycin [73]. We showed that TIMP-1 levels were profound in 0.25–0.5 U/kg > 0.1–0.05 U/kg bleomycin-treated mice. The fold increase of BALF TIMP-1 compared to other mediators of this study suggests that TIMP-1 could be one of the major contributors to fibrogenesis processes.

In clinical studies, CRP is a biomarker of declining lung function and a determinant of survival in respiratory illnesses such as COPD (Chronic Obstructive Pulmonary Disease) and interstitial lung disease. Higher CRP levels are predictive of shorter survival and declined lung function [47,74]. Therefore, in our study, we studied BALF levels of CRP as a predictive biomarker for non-invasive lung functions. We showed a dose-dependent and significant increase in CRP in 0.5 > 0.25 > 0.1 > 0.05 U/kg bleomycin-dosed mice. We also noticed that histopathology changes and score correlates with BALF CRP levels, suggesting a possible association between fibrosis severity, declined lung function, and CRP.

Lastly, we used histopathology to correlate the inflammatory and mechanistic readouts [8]. We observed pathological changes in lung tissue in response to bleomycin challenge. Histology confirmed fibrotic changes, and the modified Ashcroft scale correlates with the severity of fibrosis due to bleomycin insult [75]. Lung architecture has been preserved in mice treated with 0.05 U/kg bleomycin, similar to normal control, whereas 0.1 U/kg induces moderate fibrotic changes. The histopathology changes in 0.05 and 0.1 U/kg correlated with inflammatory and biochemical readouts. We demonstrated profound fibrotic changes, deposition of fibrotic mass in lung tissue, and fibrosis severity in lungs treated at 0.25 and 0.5 U/kg bleomycin, suggesting the increase in inflammation and biochemical parameters has been sufficient to induce detectable fibrotic pathology and severity in mice lungs. Our histopathology observations are consistent with those reported at higher concentrations of bleomycin by Wollin L et al., Kim SM et al., and Swaney J et al. [12,24,28].

This is the first study in which we provided a 60-fold concentration dose range of the bleomycin–response relationship to examine features of experimental fibrosis in a mouse IPF model. We demonstrate dose-dependent changes and a saturated fibrotic phenotype using bleomycin doses less than 1 U/kg, and more than 1 U/kg, respectively. Our investigation also covers 0% to optimum (7–8%) weight loss with recovery, to severe progressive (>15%) body weight loss without recovery. We also noted mortality due to bleomycin exposure, which is consistent with previous reports at a high dose.

In previous reports, investigators studied the dose effect of bleomycin at 1, 2, and 4 mg/kg [28], and 1.5, 3, and 5 U/kg [12] in mice to investigate fibrosis on various readouts. They found that the extent of changes in readouts was similar across all tested doses in the respective study, regardless of the unit being mg/kg or U/kg. This indicates the maximum response due to bleomycin and suggests that achieving the bleomycin-dose-dependent fibrotic phenotype is challenging. In our study of 1–2 U/kg bleomycin, we observed changes in collagen content, lung weight, and BALF biomarkers to approximately similar extents among the tested bleomycin concentrations, suggesting a maximum response due to bleomycin, which was consistent with that reported previously. In our previous study, we investigated 0.75–2 U/kg i.t doses of bleomycin on acute lung injury in rats at an early 3-day timepoint. We reported there were significant differences in the response of inflammation and soluble disease mediators such as TGF-β1, TNF-α, IL-6, TIMP-1, and WISP-1 induced by i.t. bleomycin doses of 0.75 U/kg < 1.25–2 U/kg, whereas the response induced at 1.25–2 U/kg is not significantly different in evaluated endpoints. This infers that bleomycin greater than 1 U/kg induced a saturated increase in levels of inflammation and disease mediators in rats, like we observed in the current study using a mice model at a 14-day time point [7].

In addition, at a dose of 3 U/kg, we observed mortality. Our data and previously reported data indicate that mice can withstand an insult up to certain bleomycin doses. The lung also has the capacity for collagen synthesis. Considering these limitations and mortality results, suggesting a high dose of bleomycin is not beneficial at a certain point. It can negatively impact the mice’s physiological state, leading to morbidity and mortality. This also indicates the critical importance of selecting the optimal dose of bleomycin, as well as controlled experimental conditions for model use in preclinical research. Based on our opinion and experience, if the model exhibits a severe disease phenotype, we might end up risking losing the selection of efficacious antifibrotic novel therapeutics that are under investigation. Additionally, with high mortality, we may miss out on valuable information from the animals. Despite extensive efforts and investment, these scenarios slow the advancement of ongoing IPF research. Therefore, at the preclinical testing stage using the bleomycin model, controlling the severity and mortality is achievable by understanding the relationship between bleomycin concentrations and the fibrotic phenotype. Thus, our report is invaluable and very insightful in improving the success of experimental outcomes using a bleomycin mouse model.

In a study using <1 U/kg bleomycin, we demonstrated no mortality and a dose-dependent effect on collagen and BALF biomarker readouts, which were challenging to achieve using >1 U/kg bleomycin doses. These study data demonstrate the capture and differentiation of the effect of small incremental doses of bleomycin on the phenotype, suggesting our precision and control over experimental variables of this challenging model. In our study, by reducing the doses of bleomycin treatment ≤1 U/kg, we showed that a differential effect on features of experimental fibrosis can be achieved compared to the commonly used does of 1–2 U/kg, which resulted in a saturated effect on endpoints, and no mortality was observed in bleomycin-treated animals in contrast to the effects we observed and reported with a high dose of 3U/kg bleomycin. Ideally, the model should have an optimum severity and controlled disease phenotype without mortality. This highlights the critical importance of selecting the bleomycin concentration in running the mouse model successfully without mortality and morbidity. We highly recommend understanding the dose effect of new sources, lots, or grades of bleomycin on the fibrotic phenotype to increase the chances of success with phenotypic, mechanistic, or pharmacological interventions.

Furthermore, the severe induction of disease is accompanied by progressive weight loss without recovery towards the end of the study, less physical activity, shallow breathing, and a state of being moribund after the bleomycin challenges, which are signs of the abnormal physiological state of animals. This experimental condition is not ideal for pharmacological interventions and understanding disease mechanisms. We highly recommend using a bleomycin dose range that induces optimum weight loss with recovery and reduces the stress in the animals, and does not result in reduced physical activity, shallow breathing, or moribund state after the bleomycin challenge. This will help in maintaining the satisfactory physiological state of animals while running the model, which will help increase the chances for successful, reliable assessment of the disease phenotype and drug testing in comparison to the use of bleomycin doses that induce an unacceptable abnormal physiological state in animals [8]. This is achieved by understanding the dose effect of bleomycin on the experimental phenotype.

Our observations strongly indicate an increase in the cellular pattern of BALF macrophages > lymphocytes > neutrophils, indicating a robust, optimum, controlled, and good disease phenotype in the 14-day mouse bleomycin model. Interestingly, at low doses of 0.25–0.5 U/kg bleomycin in mice, we showed a biochemically and histopathology detectable fibrotic phenotype. Although we used low concentrations of bleomycin, we successfully captured the increased levels of TGF-β, IL-6, IL-13, WISP-1, VEGF, TIMP-1, and CRP biomarkers in our model data. Our data suggest that the fibrogenesis mechanism(s) are activated at these bleomycin concentrations, and robust fibrosis is developed in mice. In this report, we also provided a framework of readouts of experimental fibrosis, such as collagen content, fibrotic mediators, and target proteins, e.g., TGF-β, IL-6, IL-13, WISP-1, VEGF, TIMP-1, and CRP, along with histology, which are valuable readouts with translational value [7,8,44]. These efforts provide insights into possible mechanisms attributed to the fibrotic phenotype caused by 0.25–5 U/kg doses of bleomycin. We highlight that bleomycin source and potency may contribute to discrepancies or disagreements. The source of bleomycin is critical while running this model. It is also difficult to investigate each source of bleomycin against each sex or species, or to conduct a comparative analysis, which is not feasible. Therefore, we emphasize that the optimal bleomycin concentration and experimentally controlled conditions are more important while running this model under laboratory conditions.

Our study has certain limitations. We did not compare different sources of bleomycin phenotypes at similar doses. In reading past reports, we found difficulties in comparing various studies. Inconsistencies in the source and quality of bleomycin (research-grade or pharmaceutical-grade) greatly impact outcomes. Furthermore, doses of bleomycin are reported with differing units (U/kg or mg/kg) without traceable stoichiometric conversions. This brings limitations when comparing findings across studies and our data with previous reports. Therefore, despite the plethora of IPF studies that have been conducted, we compared our data with only a handful of studies that, like ours, used pharmaceutical-grade bleomycin. Human IPF pathophysiology is complex. It is initiated and propagated through complicated networks of profibrotic mediators and molecular pathways [8]. Our evaluation is limited to a few target proteins. Our histology analysis is limited to H and E staining and Ashcroft scoring. The accurate % of the fibrotic area can be studied using Masson trichome staining. We suggest CRP can be included as a predictive biomarker for non-invasive lung function analysis in this model, but its validation is warranted with more studies using suitable modalities. Our study can be extended by studying the involvement of other fibrotic mediators, target proteins, and fibrotic principal pathways, along with the mechanism of fibrogenesis and disease progression, using ideal doses of bleomycin established in the current study to better reflect the network of drivers of (pro)-fibrotic mediators involved in human IPF. The approved standards and potential novel therapeutics can be evaluated with better accuracy by using controlled experimental conditions.

## 4. Materials and Methods

### 4.1. Animals

Animal experiments and procedures were approved by the Institutional Animal Care and Use Committee of PRISM (Approval Number 14-01). The 10- to 12-week-old C57BL/6J female mice weighing 18–22 g (Jackson Laboratory, Bar Harbor, ME, USA) were used after 7 days of acclimatization under pathogen-free conditions. Food and water were available ad libitum.

### 4.2. Bleomycin and Experimental Groups

We induced pulmonary fibrosis in mice with a single intratracheal challenge using pharmaceutical-grade bleomycin obtained from Zydus-Hospira Oncology Private Ltd., Gujarat, India. The bleomycin was dissolved in 1× PBS. We randomly divided the mice into ten groups, each containing five mice. We began the experiment with a bleomycin dose of over 1 U/kg, with groups treated with 1, 1.5, 2, or 3 U/kg of bleomycin or PBS. Later, we carried out a bleomycin dose–effect experiment below 1 U/kg in which mice were randomly divided into groups and treated with 0.05, 0.1, 0.25, or 0.5 U/kg of bleomycin or PBS.

### 4.3. Induction of Lung Fibrosis and Time for Endpoint Analysis

On day 0, the mice were given anesthesia with intraperitoneal (i.p.) injections of Xylazine: Ketamine (10:80 mg/kg). Mice then received a single i.t. instillation of bleomycin (0.05–3 U/kg) using a 31-gauge needle attached to a 1 mL Hamilton syringe. The control mice were only given 50 μL of PBS. After the instillation, the mice were allowed to recover from the anesthesia, kept warm, and returned to their cages with free access to food and water. The animals were monitored for health daily over the entire duration of the study. The animals were sacrificed on day 14, post-bleomycin or PBS challenge, and fibrosis-relevant endpoints were analyzed.

### 4.4. Bronchoalveolar Lavage (BAL) and Collection of BAL Fluid (BALF)

After 14 days of bleomycin or PBS challenge, the animals were euthanized with Euthasol (100–120 mg/kg) through intraperitoneal injections. A small incision was made on the skin to expose the trachea, and BAL was performed four times using a plastic cannula with 0.3 mL 1× PBS (pH = 7.4). The volumes of individual BAL aspirates were pooled.

### 4.5. Assessment of Pulmonary Inflammatory Cells

We assessed pulmonary inflammatory cell infiltration using a previously described method [44]. We mixed equal volumes of BAL fluid (BALF) and Turk’s solution and counted total leukocytes manually using a hemacytometer (Hausser Scientific, Horsham, PA, USA). The remaining fluid was centrifuged at 10,000× *g* rpm for 10 min at 4 °C, and aliquots of BALF supernatant were collected aseptically and stored at −80 °C until analysis. Cell pellets were reconstituted in rat serum and stained with Leishman solution on frosted glass slides (Leica Biosystems, Nussloch, Germany). Using a light microscope (BX2, Olympus, Tokyo, Japan) at 100× magnification, 500 cells/slide were counted. Cells were categorized based on morphology into neutrophils, lymphocytes, eosinophils, or macrophages [76].

### 4.6. Lung Harvest for Collagen Determination and Histology

Following BAL, the right lungs were collected from the animals to assess the collagen content. The lungs were washed using 1× PBS and then placed in 1 mL of PBS which contained 0.1% (*v*/*v*) of protease and phosphatase inhibitor cocktail. The harvested right lungs were then stored at a temperature of −80 °C until use. On the other hand, the left lungs were carefully harvested for histology purposes and stored in 10% neutral buffered formalin.

### 4.7. Assessment of Vascular Leakiness and Lung Parameters

Vascular leakiness and lung parameters were assessed as described previously [76]. Vascular leakiness was assessed by quantifying BALF protein content. BALF protein concentration was determined by colorimetric detection by bicinchoninic acid (BCA) assay. Absorbances were determined at 570 nm using a spectrophotometer, and proteins were calculated based on a bovine serum albumin (BSA) standard curve and expressed in mg/mL of BALF. Lung parameters were assessed by measuring right-lung weight and lung index. Briefly, after BAL, lungs were harvested, washed in 1× PBS to remove debris, blotted using tissue paper, and weighed (wet weight). Lung index was determined by dividing wet lung weight with body weight.

### 4.8. Assessment of Lung Collagen Content by Sircol Soluble Collagen Assay

Fibrosis was assessed by quantifying total soluble collagen using the Sircol collagen assay kit (Biocolor Ltd., Carrickfergus, UK), as described previously, with some modification [7,8]. Briefly, wet right lungs were washed in 1× PBS and homogenized in 1 mL of CHAPS detergent buffer. The lung homogenate was mixed with an equal volume of acid pepsin solution (5 mg/mL of 0.5 M acetic acid) and incubated overnight at 4 °C. Following centrifugation, the supernatant was assayed for soluble collagen content according to the manufacturer’s instructions. Absorbance at 555 nm was read on a VersaMax ELISA Microplate Reader (Molecular Devices, LLC., San Jose, CA, USA). Lung collagen data were expressed as µg of soluble collagen per right lung of mice [7].

### 4.9. Assessment of Profibrotic Environment of Lung

The fibrotic environment of lung fibrosis was analyzed by assessing profibrotic cytokine levels in BALF, which was determined using commercially available ELISA (enzyme-linked immunosorbent assay) according to the manufacturer’s instructions. The Quantikine ELISA kit, used for TGF-β1, IL-6, TIMP-1, VEGF, IL-13, CRP, and WISP1, was purchased from R & D Systems (Minneapolis, MN, USA) [7,8,44,76].

### 4.10. Histopathological Evaluation of Progression of Pulmonary Fibrosis

The left lungs were processed using a routine histology protocol. Paraffin-embedded tissue was sliced into 4 μm, and histology section slides were stained with Hematoxylin & Eosin (H&E). Pathological changes in lung tissue were assessed using criteria adapted from a previously published protocol by Ashcroft et al. and Hubner et al. [75,77]. The severity of fibrotic changes in each lung section was assessed as a mean score of severity [8].

### 4.11. Statistical Analysis

All data are presented as the mean ± standard error of the mean (SEM). The data were analyzed using one-way ANOVA followed by Dunnett’s test for multiple comparisons using GraphPad Prism version 9. The Goodness of Fit (*r*^2^) was used to examine statistical correlations between concentrations of bleomycin (independent variables) and features of experimental fibrosis (dependent variables) analyzed by simple linear regression analysis using GraphPad Prism version 10. Pearson correlation coefficient (*r*) values between two variables were obtained in Excel using the PEARSON function. A *p*-value < 0.05 compared with control was set as statistically significant.

## 5. Conclusions

In this report, we investigated the impact of bleomycin doses on experimental features of lung fibrosis in the mouse bleomycin model. Our careful experimental observations, accuracy, and comprehensive data set provided meaningful insights into bleomycin dose effects on the fibrotic phenotype. We present highly calibrated findings demonstrating bleomycin concentration’s dose-dependent and saturated effects on the fibrotic phenotype. At certain concentrations of bleomycin, the phenotype was saturated, and further increases in dose induced mortality. Therefore, the ideal bleomycin concentration for the model should be chosen appropriately to provide a controlled fibrotic phenotype and aid in the reliable assessment of fibrotic features. This report discusses various concentrations of bleomycin that induce no fibrosis, fibrosis without mortality, and 100% mortality. The insights of the current report are valuable for early-stage preclinical development of anti-fibrotic compounds, guiding experimental design, endpoints, and biomarker selection, and helping achieve specific experimental objectives such as therapeutic interventions, understanding mechanisms of action, and conducting survival studies during the active phase of the bleomycin model. Although our work is limited to a set of parameters, insights from this report can be useful in increasing the utility of the mouse bleomycin model in translational lung fibrosis research.

## Data Availability

All relevant data are within the manuscript. Further inquiries can be directed to the corresponding author.

## Supplementary Materials

The following supporting information can be downloaded at: https://www.mdpi.com/article/10.3390/ijms252212300/s1.
