# Supplementary material for: Highly Calibrated Relationship Between Bleomycin Concentrations and Facets of the Active Phase Fibrosis in Classical Mouse Bleomycin Model
Source: Int J Mol Sci. 2024 Nov 15;25(22):12300. doi: 10.3390/ijms252212300 (PMC11595013; doi:10.3390/ijms252212300)
Supplement: Supplementary file 1 [file ijms-25-12300-s001.zip › ijms-3282742-supplementary.pdf]

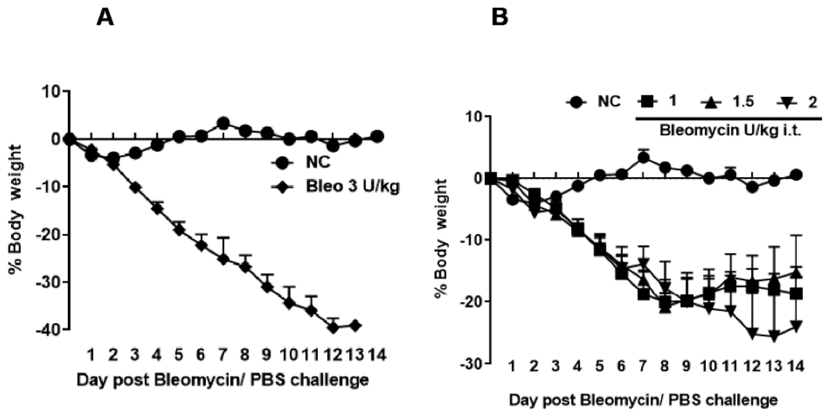

Supplementary Figure S1: Effect on % body weight change from day 0 to 14 due to 1-3 U/kg bleomycin i.t. challenge. Note: This data represents the weight loss of all animals until they were alive during the study duration. In 3 U/kg bleomycin-treated groups, animals died in the middle of the study. n=5 mice/group

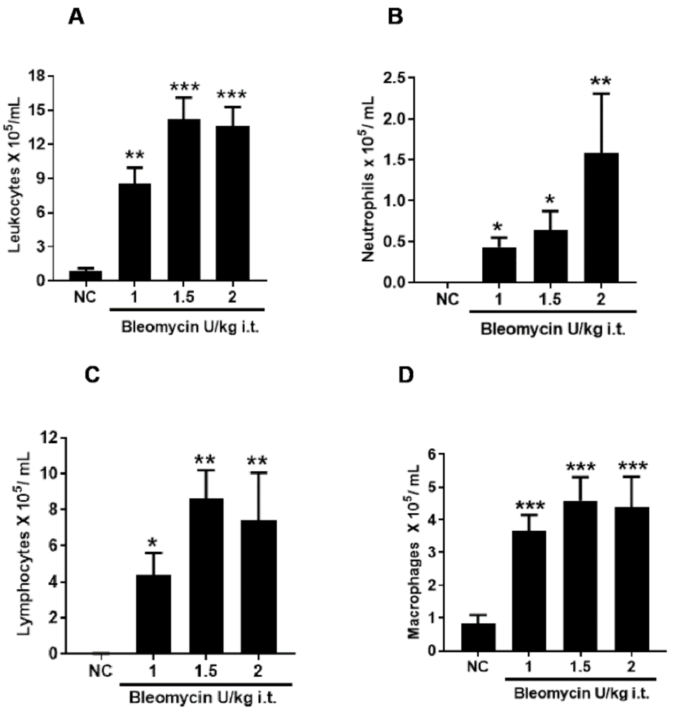

Supplementary Figure S2: Effect of 1-2 U/kg intratracheal bleomycin concentrations on pulmonary inflammation. (**A)** leukocytes (TLC), **(B)** macrophages, **(C)** lymphocytes, and **(D)** neutrophils. Data are expressed as mean ±SEM of n=5 mice/group; *p <0.05; **p<0.01; and ***p<0.001 Vs NC

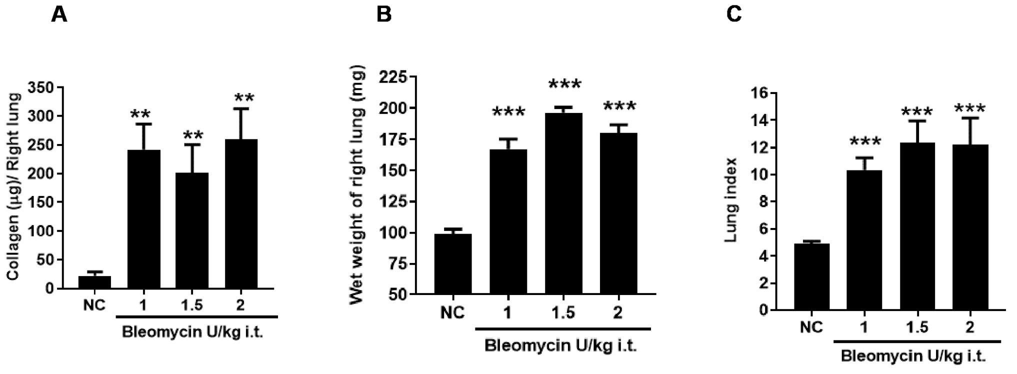

Supplementary Figure S3: Effect of 1-2 U/kg intratracheal bleomycin concentrations on lung parameters. **(A)** right lung collagen content, **(B)** wet weight of right lung, **(C)** lung index. Data are expressed as mean ±SEM of n=5 mice/group. *p <0.05; **p<0.01; and ***p<0.001 Vs NC.

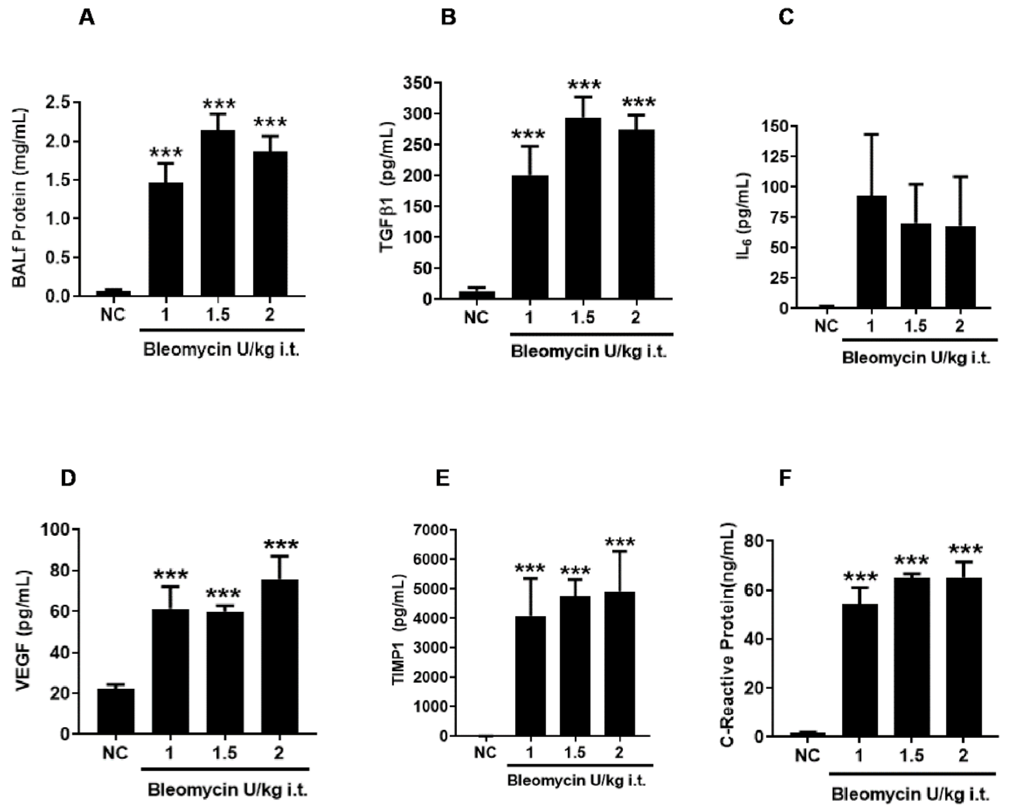

Supplementary Figure S4: Effect of 1-2 U/kg intratracheal bleomycin concentrations on
BALF lung fibrosis biomarkers. **(A)** BALF protein content, **(B)** TGFβ-1, **(C)** IL-6, **(D)** VEGF, **(E)** TIMP-1, **(F)** c-Reactive protein. Data are expressed as mean ± SEM of n=5 mice/group. *p<0.05; **p<0.01; and ***p<0.001 Vs NC.

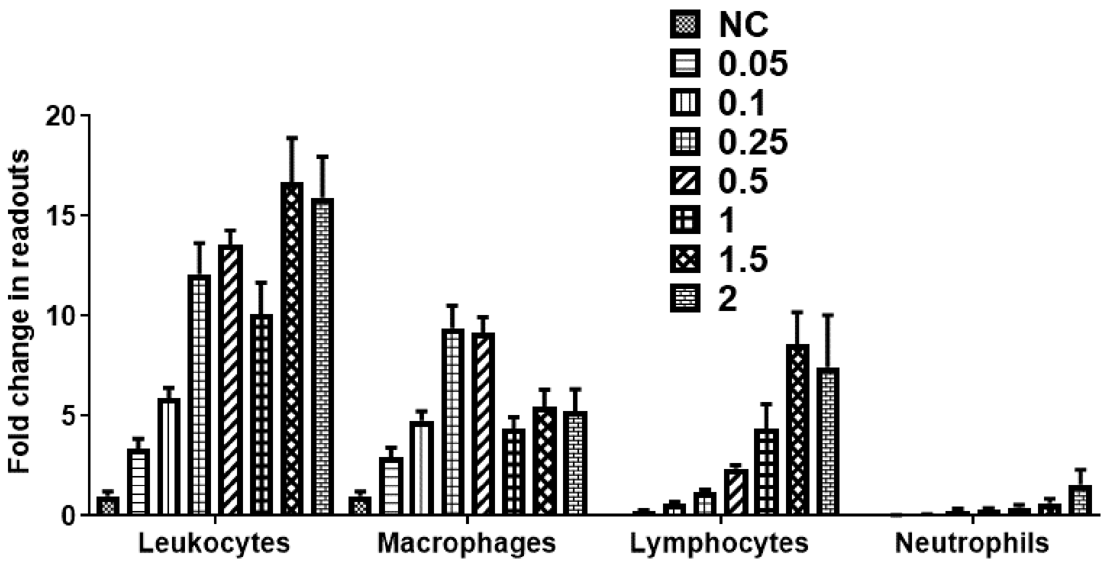

Supplementary Figure S5. Effect of 0.05-2 U/kg bleomycin challenge on fold change in inflammatory cell infiltration of leukocytes, macrophages, lymphocytes*, and neutrophil* due to challenge of 0.05-2 U/kg bleomycin in mice on day 14. (* actual levels).

Supplementary Table S1: Summary of bleomycin source, sex, and unit used to induce experimental fibrosis in mice.

Sr. No	Mice strain	Bleomycin grade	Sex	Source	Concentration	Bleomycin challenge route	Reference
1	C57BL/6	Pharmaceutical grade	F	Nippo Kayko Japan	125 mg/kg	O.A.	[1]
2	C57BL/6			Nippon Kayaku (Tokyo).	5 mg/kg	i.t.	[2]
3	ICR		M	Nippon Kayaku, Tokyo, Japan	1, 2, 4 mg/kg	i.t.	[3]
4	C57BL/6		F	Nippon Kayaku, Tokyo, Japan	5 mg/kg	i.t.	[4]
5	C57BL/6		F	Nippon Kayaku, Tokyo, Japan	2 mg/mouse/d;	I.P. Days 1 and 8	[5]
6	C57BL/6		F	Nippon Kayaku, Tokyo, Japan	(0.02 or 0.03 units/mouse)	i.t.	[6]
7	C57BL/6		F	Teva Pharma Israel	2 U/kg	OA	[7]
8	**C57BL/6**		Not mentioned	Teva pharma	1 U/kg	i.t.	[8]
9	C57BL/6		F	Bellon; Sanofi-Aventis, France	3 mg/kg	i.n.	[9]
10	C57BL/6		F	(Beijing Solarbio Science & Technology Co. Ltd., Beijing, China)	5 mg/kg	i.t.	[10]
11	C57BL/6		F	Hospira, Lake Forest.	1.5 U/kg	OA	[7]
12	C57BL/6		M	Hospira, Lake Forrest	1.5 U/kg	OA	[11]
13	C57BL/6		F	Hospira (Lake Forest)	1.5 U/kg and 2.5 U/kg	i.t.	[12]
14	C57BL/6		M	Hospira Healthcare Corp., Saint-Laurent,	0.06 U/mouse (3 U/kg)	i.t.	[13]
15	C57BL/6		F	Selleckchem, USA	3 U/kg	i.t.	[14]
16	C57BL/6		Not mentioned	APP Pharmaceuticals, LLC	1.0–1.5 IU/kg	i.t.	[15]
17	C57BL/6		F	APP Pharmaceuticals, Schaumburg, IL)	0.005 U/g	i.t.	[16]
18	C57BL/6		M	MP Biomedicals, Solon, OH, USA	1-5 U/kg or repetitive challenge time course study, animals were administered bleomycin (2 U/kg) every other week for a total of 8 doses	i.t.	[17]
19	C57BL/6		F	Pharmacia, Kalamazoo, MI)	1.0 U/ml	i.t.	[18]
20	C57BL/6	Research grade	F	Sigma (St. Louis)	5 mg/kg	i.t.	[19]
21	C57BL/6		M	Calbiochem, Darmstadt, Germany	0.5mg/kg	i.t.	[20]
22	C57BL/6		F	EMD-Calbiochem	3 U/kg	i.t.	[21]
23	C57BL/6		M	Calbiochem, Darmstadt, Germany	0.25, 0.5, 0.75 and 1 mg/kg. (1±4 U/mg)	i.t.	[22]

OA:oropharyngeal aspiration, i.t.: Intratracheal; i.n.: Intranasal, i.p. : intraperitoneal

Supplementary Table S2: List of compounds evaluated in bleomycin-induced lung fibrosis in female C57BL/6 mice and successfully advanced into clinical trials for the IPF.

Sr. No	Molecule and IPF Clinical Target	Current advanced stage of clinical development	Preclinical evaluations of compounds for their antifibrotic effect in Female C57BL/6 mice			
			**Mice and sex**	**Bleo Route**	**Mechanism of actions explored**	**References**
**1**	Galectin 3 inhibitor/ TD139/GB0139	Phase IIb GALACTIC-1 Trial of GB0139	Female C57BL/6 mice	i.t.	TD139 blocked TGF-β–induced β-catenin activation in vitro and in vivo and attenuated the late-stage progression of lung fibrosis after bleomycin	[23]
**2**	AM966 LPA receptor 1 antagonist and Pirfenidone	LPA1 antagonist: NCT06003426;<br><br>Pirfenidone: Approved for IPF	Female C57BL/6 mice	i.t.	On day 3 : total protein, inflammatory cells, and LDH activity. On day 7: total protein, collagen, total (TGFβ1), Representative histopathological, and Total cellularity. On day 14: Total protein, LDH activity (cell death marker) and concentrations of the pro-fibrotic factors , TIMP-1, TGFβ1, hyaluronan and MMP-7	[24]
**3**	Nintedanib	Approved for IPF	Female C57BL/6 mice	i.t.	Fibrosis by histology, Total collagen, IL-1β, IL-6, TIMP-1, inflammatory cells	[9]
**4**	Vitamin D	Phase II NCT03770845	Female C57BL/6 mice	i.t.	Relative change in collagen1a1, (col3a1), alpha-smooth muscle actin (a-SMA) and vitamin-D receptor (Vdr) mRNA levels, Fibrosis by histology	[25]
**5**	Human serum amyloid P (SAP) / Recombinant human pentraxin-2 therapy/ PRM-151	NCT04594707	Female C57BL/6 mice	i.t.	hydroxyproline analysis, procollagen Ial and procollagen III gene expression, gene transcript levels determined by branched DNA technology at day 21 for NOS2, CCL2, oncostatin (OSM) and MARCO , and FIZZ1 and ST2	[26]
**6**	Nintedanib, and Pirfenidone	Approved for IPF	Female C57BL/6 mice	i.t.	(A) Levels of TGF-β1, SP-A, SP-D and KL-6 in the plasma of mice. α-SMA and collagen I , protein expression levels of Fn, TGF-β-R2, α-SMA, p-JAK2, JAK2, p-STAT3, and STAT3and Histology	[27]
**7**	Lysyl oxidase 2 Inhibitor	Phase II	Female C57BL/6 mice	i.t	Ashcroft scoring, hydroxyproline content, ol1a1, Col3a1, Col4a1, and Fn mRNA expression, α-SMA expression, BALF TGF-β level, Pai1 and Ctgf Mrna, Body weights, Lung Histology	[28]
**8**	Treprostinil/BW245C / D Prostanoid Receptor Agonist	Phase III Recruiting (NCT04708782)	Female C57BL/6 mice	transorally	lung inflammation, fibronectin, hyaluronic acid, OH-proline in lung, and soluble collagen	[29]
**9**	Anlotinib/ Multitargeted tyrosine kinase inhibitor	Phase III (NCT05828953)	Female C57BL/6 mice	i.t.	Lung function, Hydroxyproline (HYP, α-SMA and Fibronectin, Histology	[30]
**10**	PBI-4050/G protein–coupled receptors with free fatty acid ligands	Phase II and Phase III	Female C57BL/6 mice	i.t.	Col-I, Fibronectin, PAI-1, TIMP-1, GMCSF, IL13, MCP1,	https://www.atsjournals.org/doi/epdf/10.1164/ajrccm-conference.2019.199.1_MeetingAbstracts.A7231?role=tab

References

1. Makino, H., et al., *Antifibrotic effects of CXCR4 antagonist in bleomycin-induced pulmonary fibrosis in mice.* J Med Invest, 2013. **60**(1-2): p. 127-37.
2. Tao, L., et al., *Mogroside IIIE, a Novel Anti-Fibrotic Compound, Reduces Pulmonary Fibrosis through Toll-Like Receptor 4 Pathways.* J Pharmacol Exp Ther, 2017. **361**(2): p. 268-279.
3. Kim, S.N., et al., *Dose-response Effects of Bleomycin on Inflammation and Pulmonary Fibrosis in Mice.* Toxicol Res, 2010. **26**(3): p. 217-22.
4. Li, X., et al., *NMDA receptor activation inhibits the antifibrotic effect of BM-MSCs on bleomycin-induced pulmonary fibrosis.* Am J Physiol Lung Cell Mol Physiol, 2018. **315**(3): p. L404-L421.
5. Tabata, C., et al., *All-trans-retinoic acid prevents radiation- or bleomycin-induced pulmonary fibrosis.* Am J Respir Crit Care Med, 2006. **174**(12): p. 1352-60.
6. Murakami, S., et al., *Prostacyclin agonist with thromboxane synthase inhibitory activity (ONO-1301) attenuates bleomycin-induced pulmonary fibrosis in mice.* Am J Physiol Lung Cell Mol Physiol, 2006. **290**(1): p. L59-65.
7. Kulkarni, A.A., et al., *The triterpenoid CDDO-Me inhibits bleomycin-induced lung inflammation and fibrosis.* PLoS One, 2013. **8**(5): p. e63798.
8. Reed, E.B., et al., *Anti-fibrotic effects of tannic acid through regulation of a sustained TGF-beta receptor signaling.* Respir Res, 2019. **20**(1): p. 168.
9. Wollin, L., et al., *Antifibrotic and anti-inflammatory activity of the tyrosine kinase inhibitor nintedanib in experimental models of lung fibrosis.* J Pharmacol Exp Ther, 2014. **349**(2): p. 209-20.
10. Zhao, Y., et al., *Human Endometrial Regenerative Cells Attenuate Bleomycin-Induced Pulmonary Fibrosis in Mice.* Stem Cells Int, 2018. **2018**: p. 3475137.
11. Judge, J.L., et al., *Prevention and treatment of bleomycin-induced pulmonary fibrosis with the lactate dehydrogenase inhibitor gossypol.* PLoS One, 2018. **13**(5): p. e0197936.
12. Zhao, H., et al., *Pulmonary delivery of docosahexaenoic acid mitigates bleomycin-induced pulmonary fibrosis.* BMC Pulm Med, 2014. **14**: p. 64.
13. Mukherjee, S., et al., *Disruption of Calcium Signaling in Fibroblasts and Attenuation of Bleomycin-Induced Fibrosis by Nifedipine.* Am J Respir Cell Mol Biol, 2015. **53**(4): p. 450-8.
14. Shi, Y., et al., *Distal airway stem cells ameliorate bleomycin-induced pulmonary fibrosis in mice.* Stem Cell Res Ther, 2019. **10**(1): p. 161.
15. Liu, Q., et al., *IL-33-mediated IL-13 secretion by ST2+ Tregs controls inflammation after lung injury.* JCI Insight, 2019. **4**(6).
16. Heidari, B., *The importance of C-reactive protein and other inflammatory markers in patients with chronic obstructive pulmonary disease.* Caspian J Intern Med, 2012. **3**(2): p. 428-35.
17. Peng, R., et al., *Bleomycin induces molecular changes directly relevant to idiopathic pulmonary fibrosis: a model for "active" disease.* PLoS One, 2013. **8**(4): p. e59348.
18. Fujita, M., et al., *Doxycycline attenuated pulmonary fibrosis induced by bleomycin in mice.* Antimicrob Agents Chemother, 2006. **50**(2): p. 739-43.
19. Sun, L., et al., *A Chinese Traditional Therapy for Bleomycin-Induced Pulmonary Fibrosis in Mice.* Can Respir J, 2018. **2018**: p. 8491487.
20. Herrmann, F.E., et al., *Olodaterol shows anti-fibrotic efficacy in in vitro and in vivo models of pulmonary fibrosis.* Br J Pharmacol, 2017. **174**(21): p. 3848-3864.
21. Cai, Y., et al., *Transgenically-expressed secretoglobin 3A2 accelerates resolution of bleomycin-induced pulmonary fibrosis in mice.* BMC Pulm Med, 2015. **15**: p. 72.
22. Gilhodes, J.C., et al., *Quantification of Pulmonary Fibrosis in a Bleomycin Mouse Model Using Automated Histological Image Analysis.* PLoS One, 2017. **12**(1): p. e0170561.
23. Mackinnon, A.C., et al., *Regulation of transforming growth factor-beta1-driven lung fibrosis by galectin-3.* Am J Respir Crit Care Med, 2012. **185**(5): p. 537-46.
24. Swaney, J.S., et al., *A novel, orally active LPA(1) receptor antagonist inhibits lung fibrosis in the mouse bleomycin model.* Br J Pharmacol, 2010. **160**(7): p. 1699-713.
25. Tzilas, V., et al., *Vitamin D prevents experimental lung fibrosis and predicts survival in patients with idiopathic pulmonary fibrosis.* Pulm Pharmacol Ther, 2019. **55**: p. 17-24.
26. Murray, L.A., et al., *Serum amyloid P therapeutically attenuates murine bleomycin-induced pulmonary fibrosis via its effects on macrophages.* PLoS One, 2010. **5**(3): p. e9683.
27. Yang, Y., X. Wang, and J. Zhang, *Pirfenidone and nintedanib attenuate pulmonary fibrosis in mice by inhibiting the expression of JAK2.* J Thorac Dis, 2024. **16**(2): p. 1128-1140.
28. Cheng, T., et al., *Lysyl oxidase promotes bleomycin-induced lung fibrosis through modulating inflammation.* J Mol Cell Biol, 2014. **6**(6): p. 506-15.
29. van den Brule, S., et al., *The D prostanoid receptor agonist BW245C [(4S)-(3-[(3R,S)-3-cyclohexyl-3-hydroxypropyl]-2,5-dioxo)-4-imidazolidineheptanoic acid] inhibits fibroblast proliferation and bleomycin-induced lung fibrosis in mice.* J Pharmacol Exp Ther, 2010. **335**(2): p. 472-9.
30. Chen, W., et al., *Anlotinib Inhibits PFKFB3-Driven Glycolysis in Myofibroblasts to Reverse Pulmonary Fibrosis.* Front Pharmacol, 2021. **12**: p. 744826.
